# Doxorubicin and etoposide sensitize small cell lung carcinoma cells expressing caspase-8 to TRAIL

**DOI:** 10.1186/1476-4598-9-87

**Published:** 2010-04-23

**Authors:** Alena Vaculova, Vitaliy Kaminskyy, Elham Jalalvand, Olga Surova, Boris Zhivotovsky

**Affiliations:** 1 Institute of Environmental Medicine, Division of Toxicology, Karolinska Institutet, Box 210, SE-171 77 Stockholm, Sweden

## Abstract

**Background:**

TRAIL is considered as a promising anti-cancer agent, because of its ability to induce apoptosis in cancer but not in most normal cells. However, growing evidence exist that many cancer cells are resistant to its apoptotic effects. SCLC is a typical example of tumor entity where TRAIL monotherapy is not efficient.

**Results:**

We demonstrated that doxorubicin and etoposide markedly sensitized SCLC cells expressing caspase-8 to apoptotic effects of TRAIL. The drug-mediated sensitization of these cells was associated with increase of surface and total DR5 protein level, specific cleavage of cFLIP_L_, decrease of cFLIP_S _level, and a strong activation of caspase-8. The involvement of mitochondria-mediated pathway was demonstrated by enhanced Bid cleavage, Bax activation, and cytochrome *c *release. Activation of caspase-8 induced by combined treatment was shown to occur upstream of mitochondria and effector caspases.

**Conclusions:**

Our results highlight significant applicability of doxorubicin and etoposide in sensitization of SCLC cells expressing caspase-8 to treatment with TRAIL.

## Background

Lung cancer (LC) is a major cause of cancer deaths in the Western world. Based on the histo-pathological features, LC is divided into small cell lung carcinoma (SCLC), and non-small cell lung carcinoma (NSCLC), which account for 25 and 75% of bronchogenic carcinomas, respectively. In contrast to NSCLC, SCLC is characterized by relatively high sensitivity to treatment with anticancer drugs and radiation. However, despite the initial responsiveness, relapses occur in most cases, accompanied by the fast development of severe resistance to treatments during the course of disease. SCLC represents a highly malignant and particularly aggressive form of cancer, with early and widespread metastases, and poor prognosis. Mechanisms responsible for the intrinsic and acquired resistance to treatment involve the defects/dysregulations of the apoptotic programme [1,2]. The avoidance of apoptosis is considered as one of the hallmarks of cancer cells, and represents a significant clinical problem. Therefore, elucidation of the mechanisms and molecules responsible for the resistance is essential for proper targeting of anticancer therapy.

The tumour necrosis factor (TNF)-related apoptosis-inducing ligand (TRAIL), a member of TNF family, is particularly interesting because of its unique ability to induce cancer cell death while sparing the most of normal cells. This implies its potential promise as an anti-cancer agent [3]. TRAIL can interact with different receptors. Only two of them, namely, death receptors (DR) contain apoptosis-related death domain (DD): DR4 (TRAIL-R1) and DR5 (TRAIL-R2). Decoy receptors DcR1 (TRAIL-R3) and DcR2 (TRAIL-R4) either lack or posses truncated DD and are, therefore, not able to transmit apoptotic signal. Osteoprotegerin (OPG, TRAIL-R5) is a soluble receptor with the lowest afinity to TRAIL [4]. TRAIL binding to DR4 and DR5 results in triggering of the extrinsic pathway, initiated by formation of the death-inducing signalling complex (DISC) consisting of Fas-associated DD protein (FADD) and pro-caspase-8. Activation of caspase-8 at the DISC level plays a crucial role in the DR-mediated pathway, and can be efficiently regulated by its competitive inhibitor cFLIP (FLICE-like inhibitory protein). Caspase-8 activation is followed by cleavage of effector caspases and apoptosis execution (characteristic for type I cells). In some cases, caspase-8 can also cleave Bid, which is responsible for translocation of apoptotic signal to mitochondria. Subsequent amplification of the death signal at the level of these organelles is essential in so-called type II cells [5,6].

TRAIL triggers apoptosis in a broad spectrum of cancer cell lines *in vitro *and *in vivo *[7,8]. However, failure to undergo apoptosis in response to TRAIL has been demonstrated in majority of SCLC cells [9,10]. Significant perturbances of apoptosis programme such as downregulation/absence of some proapoptotic proteins and/or overexpression of anti-apoptotic proteins have been shown to be a characteristic feature of SCLC cells [11]. The higher rates of loss of expression of caspase-8, caspase-10, DR4, DR5, Fas, and FasL have been found in SCLC compared to NSCLC cells [9,12]. A relationship between the inactivation of some DISC components and Myc oncogene amplification, which is a common event in SCLC, has also been reported [9].

Majority of chemotherapeutic agents are typical activators of mitochondria-mediated (intrinsic) apoptotic pathway, where release of cytochrome *c *from mitochondrial intermembrane space is followed by formation of apoptosome complex (cytochrome *c*, Apaf-1, dATP, pro-caspase-9), activation of initiator caspase-9 and downstream effector caspases. The described events can be effectively modulated by pro-apoptotic (*e.g*. Bid, Bax, Bak) and/or anti-apoptotic (*e.g*. Bcl-2, Mcl-1, Bcl-X_L_) members of Bcl-2 family [13]. Caspase-2 has been shown as an important link between DNA damage and the engagement of the mitochondrial pathway [14].

The clinically relevant concentrations of chemotherapeutic drugs might restore the apoptotic response to TRAIL in various cancer cells through different mechanisms and, therefore, sensitize these cells to TRAIL treatment. Among them, upregulation of the DRs, facilitation of DISC formation, downregulation of anti-apoptotic proteins, enhancement of activation of mitochondrial pathway and caspase cascade are particularly interesting [15,16]. However, respective data concerning SCLC treatments are still missing. The usual therapeutic regimes used for this type of cancer include *e.g*. doxorubicin, etoposide or cisplatin. As TRAIL monotherapy has been shown to be ineffective in SCLC, in present study we explored the potential use of combination of TRAIL and doxorubicin or etoposide in order to provide a tool for triggering apoptosis in resistant cancer cells.

## Materials and methods

### Cell culture and treatments

Human SCLC cell lines - H69 (ECACC), H82 (ATCC), U1285, U1690, and U1906 [17] were grown in RPMI 1640 medium supplemented with 10% of heat-inactivated fetal bovine serum (FBS), glutamine (2 mM), penicillin (100 U/ml) and streptomycin (100 μg/ml) (all from Gibco) in 37°C, 5% CO_2 _and 95% humidity. Twenty-four hours after seeding, cells were treated with human recombinant Killer TRAIL (provided by Dr. L. Andera), doxorubicin (Sigma), etoposide (Vepesid, Bristol-Myers), PKC412 (CGP41251, Novartis), cycloheximide (Sigma), pan-caspase inhibitor z-VAD-fmk (Enzyme Systems Products) or vehicle (DMSO) for the time points and concentrations indicated in figure legends.

### Surface TRAIL receptor expression

The level of surface TRAIL receptors was detected in cells after incubation with specific antibodies using flow cytometry [18]. Briefly, after washing with PBS + 5% FBS, cells were incubated (20 minutes, 4°C) with primary antibody (anti-TRAIL-R1 to -4 flow cytometry set, Axorra; HS101, HS201, HS301, HS402 antibodies, respectively). Cells were then washed twice, and incubated (20 min, 4°C) with secondary antibody (FITC-conjugated goat anti-mouse IgG1, ALX-211-200, Axorra or AlexaFluor-488-conjugated donkey anti-mouse-IgG, A21202, Molecular Probes). After washing twice, the cells were stained (15 min, 4°C) with 7-AAD (1 μg/ml, Molecular Probes) and analyzed using flow cytometry (FACScan, Becton Dickinson). The 7-AAD negative cells were subjected to receptor analysis (Cell Quest software). Results are expressed as histograms (green fluorescence indicating the amount of the receptor present at the cell surface versus cell counts), and related to appropriate control lacking the specific primary antibody.

### Analysis of Bax activation

Cells were washed in PBS, fixed in 1% paraformaldehyde (10 minutes, 4°C), washed again, and incubated (30 min, 4°C) with mouse anti-Bax antibody (BD556467, Becton Dickinson) diluted in PBS containing 1% BSA and 0.1% saponin. After additional washing, cells were incubated (30 min, 4°C) with AlexaFluor-488-conjugated donkey anti-mouse IgG secondary antibody (A21202, Molecular Probes) diluted in PBS + 1% BSA + 0.1% saponin, washed twice, and resuspended in PBS. For each sample, controls lacking the specific primary antibody were prepared. Analysis was performed using flow cytometer (FACScan, Becton Dickinson) and CellQuest software. Ten thousand cells per sample were analysed and the results were expressed as percentage of cells with active Bax.

### Analysis of mitochondrial membrane potential (MMP)

After washing in PBS, cells were incubated (20 minutes) in HBSS with 25 nM of tetramethylrhodamine ethyl ester perchlorate (TMRE, Molecular Probes), washed twice with HBSS, and analysed (2 × 10^4 ^cells per sample) by flow cytometry (FACScan, Becton Dickinson). Forward and side scatters were used to gate the viable cell population. The data were evaluated using Cell Quest software as a percentage of the cells with decreased MMP.

### Caspase activity assay

Cells were lysed in appropriate lysis buffer, incubated with caspase-3, caspase-8 or caspase-2 substrates and analysed as described previously [19]. The values of caspase activity were related to the total protein amount. Caspase activity was expressed as a fold-increase to appropriate control.

### Immunoblotting

Cells were lysed in Complete lysis buffer (Roche) with protease inhibitor cocktail (PIC, Complete-M, Roche). The protein concentration was determined in samples (BCA protein assay, Pierce), which were mixed with Laemmli buffer and subjected to SDS-PAGE and western blotting [19]. For immunodetection, following antibodies were used: anti-cleaved PARP (CS9546), anti-cleaved lamin A (CS2036), anti-cleaved caspase-3 (CS9661), anti-Bid (CS2002) (Cell Signaling Technology), anti-DR4 (D3813), anti-DR5 (D3938), anti-Mcl-1 (M8434) (Sigma), anti-caspase-10 (M059-3, MBL), anti-FLIP (NF6, 804-428, Axorra), anti-caspase-2 (BD611022), anti-FADD (BD556402), anti-cytochrome *c *(BD556433), and anti-Bax (BD556467) (Becton Dickinson), anti-survivin (ab469, Abcam), anti-caspase-8 (provided by Prof. P. Krammer), anti-Bcl-2 (sc492), anti-Bcl-X_L _(sc-634) (Santa Cruz Biotechnology). The recognized proteins were detected using horseradish peroxidase-labelled secondary antibodies: anti-mouse IgG (31430, Pierce), anti-rabbit IgG (31460, Pierce), and enhanced chemiluminescence kit (Western blot detection reagent, GE Healthcare UK Limited). An equal loading was verified using anti-β-actin (A2066, Sigma), anti-G3PDH (2275, Trevingen) or anti-TOM40 (sc-11414, Santa Cruz Biotechnology, for mitochondrial fraction) antibodies. For detection of cytosolic and mitochondrial cytochrome *c *and Bax, the cells were washed twice with PBS, incubated (5 min, 4°C) in buffer (250 mM sucrose, 70 mM KCl, 100 μg/ml digitonin in PBS), centrifuged (5 min, 7000 g), and the supernatant was collected (cytosolic fraction). Mitochondrial fraction was prepared by lysis of the pellet in Complete Lysis-M buffer with PIC (Roche).

### cFLIP and caspase-8 overexpression

Twenty four hours after seeding, cells were transfected with DNA:Lipofectamine 2000 (Invitrogen) mixture at ratio 2 μg:1 μl according to manufacturer's instructions. Following next 24 h, medium was exchanged and the cells were treated (8 or 24 h) with TRAIL (100 ng/ml), doxorubicin (1 μM) or their combination. The following plasmids were used: pcDNA3 (Invitrogen), pcDNA3-Flag-cFLIP_S _(provided by Prof. P. Krammer and Dr I. Lavrik), pcDNA-MACH alpha1 (provided by Prof. D. Wallach), pMSCV-IRES-GFP, and cFLIP_L_-pMSCV-IRES-GFP (provided by Dr A. Grandien).

### cFLIP and caspase-8 siRNA experiments

Three different specific siRNAs were used to downregulate (a) both long and short cFLIP, (b) the long form, or (c) the short form only. The non-targeting siRNA was used as control. All the siRNAs were synthesized by Qiagen according to Galligan et al. [20]. The siRNA was diluted in 100 μl of Opti-MEM I medium (Gibco), and 1 μl of Lipofectamine 2000 reagent (Invitrogen) was added to other 100 μl of OptiMEM I medium. Diluted siRNA and Lipofectamine 2000 were then mixed and incubated for 20 min. Transfection complexes were added (200 μl per well) to cells 24 h after seeding in 12 well plates. The final concentration of siRNA was 100 nM. After 24 h, the medium was exchanged, and cells were treated (24 h) with TRAIL (100 ng/ml). For caspase-8 siRNA transfection, the same experimental protocol was used, and the cells were treated with TRAIL and/or doxorubicin 48 h after transfection. Caspase-8 siRNA (L-003466-00) and control siRNA (D-001810-10) were obtained from Dharmacon.

### MTS assay

Cells were seeded in 96-well plates at a density of 10^4 ^cells per well and 24 h later treated with various concentrations of the drugs. After next 24 h, CellTiter 96 AQueous MTS Reagent Solution (Promega) was added to each well according to the manufacturer's instructions, and in 1 h the cell viability was determined by measuring the absorbance at 490 nm using Labsystems Multiscan MCC/340 plate reader.

### Analysis of nuclear morphology

Cells were washed in PBS, fixed in ethanol (70%), stained (30 min) with 4,6-diamidino-2-phenyl-indole (DAPI, Fluka, 1 μg DAPI/ml ethanol) and mounted in Mowiol. The percentage of apoptotic cells (with chromatin condensation and fragmentation) was calculated from a total number of 200 cells using LSM 510 META spectral laser scanner microscope (Zeiss).

### Analysis of DNA content

For DNA analysis, cells were fixed in 70% cold ethanol overnight, washed, incubated 1 h at 37°C with RNAse A (100 μg/ml), and exposed to PI (50 μg/ml) in phosphate-buffered saline (pH 7.4) for 30 min. The cells were analyzed by flow cytometry (FACScan, Becton Dickinson) and CellQuest software. The cell death was monitored by evaluation of percentage of cells with subdiploid amount of DNA.

### Statistical evaluation

The results of three independent experiments were expressed as the means ± S.E.M. Statistical significance (p < 0.05) was determined by one-way ANOVA followed by Tukey test or by non-parametric Mann-Whitney test.

## Results

### SCLC cell lines are resistant to TRAIL-induced apoptosis, and differ in the expression of proteins involved in TRAIL signaling

**Figure 1:**
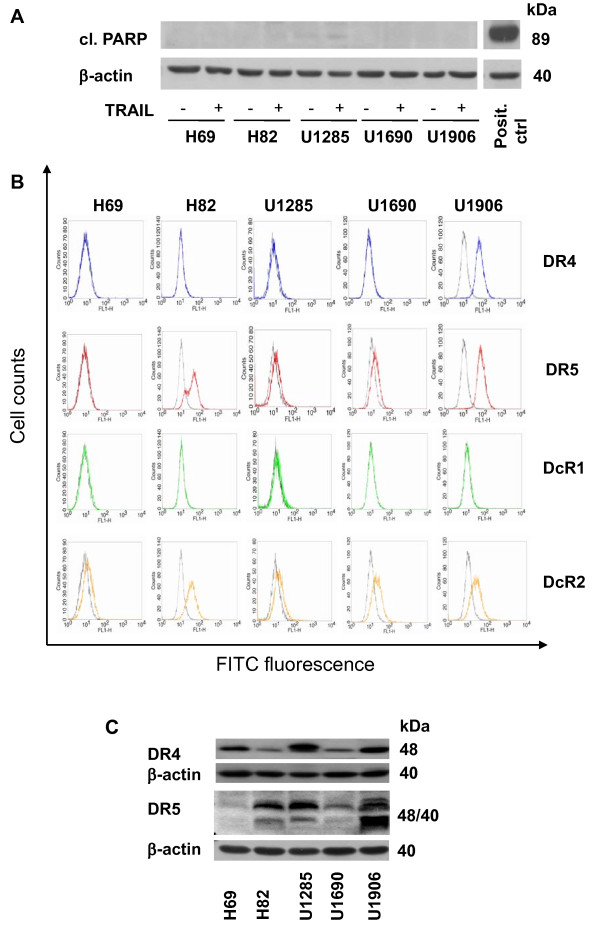
**SCLC cell lines are resistant to TRAIL treatment and differ in expression of TRAIL receptors**. (A) Cleavage of PARP was analyzed in H69, H82, U1285, U1690, and U1906 cells treated (72 h) with TRAIL (100 ng/ml). An equal loading was verified using anti-β-actin antibody. (B) Surface expression of DR4, DR5, DcR1, and DcR2 in these cells (flow cytometry, for details see Materials and methods, black lines - controls lacking primary antibody). (C) Total level of DR4 and DR5 in these cells. An equal loading was verified using anti-β-actin antibody. All results are representative of three independent experiments.

**Figure 2:**
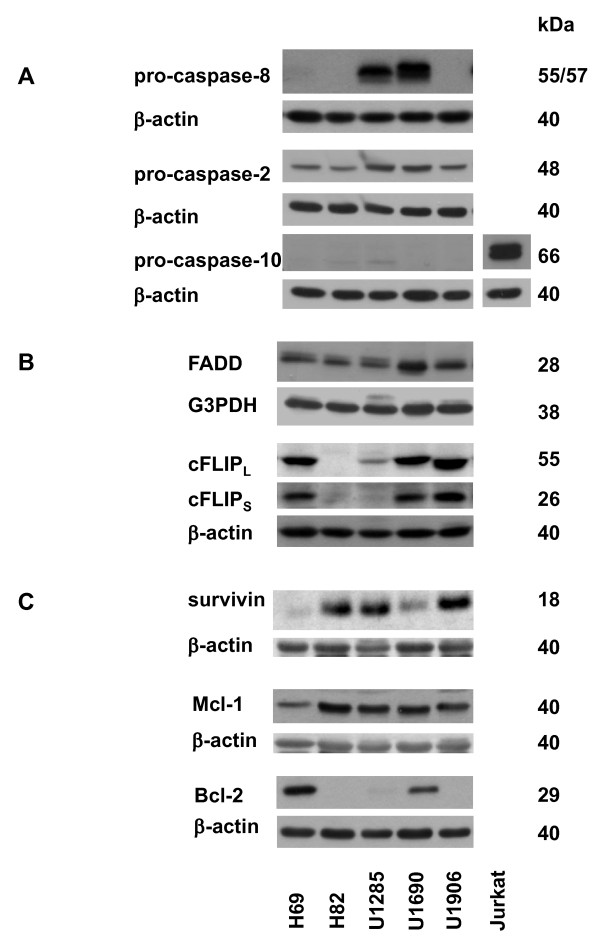
**SCLC cell lines significantly differ in expression of proteins involved in TRAIL signaling**. The level of (A) pro-caspase-8, -2, -10, (B) FADD, cFLIP_L_, cFLIP_S_, and (C) survivin, Mcl-1, and Bcl-2 proteins in H69, H82, U1285, U1690, and U1906 cells. An equal loading was controlled using anti-β-actin antibody. Jurkat cells were used as a positive control for expression of caspase-10. Results are representative of three independent experiments.

All five SCLC cell lines studied - H69, H82, U1285, U1690, U1906 - were resistant to apoptotic effects of TRAIL (100 ng/ml), as demonstrated by the absence of PARP cleavage (Figure 1A) or caspase-3 processing (not shown) after 72 h of treatment. Expression of selected proteins important in TRAIL signaling was analyzed in untreated cell lines. We showed that only 1 (U1906) out of 5 cell lines expressed DR4 at the surface, and 4 out of 5 cell lines (except for H69) did express DR5. While no surface DcR1 was present in any of cell lines, DcR2 was always detected (flow cytometry, Figure 1B). Despite its absence at the cell surface, all cell lines expressed (at various amounts) DR4 protein. The significant differences at the level of total DR5 protein were apparent among cell lines, being highest in U1906, not detectable in H69, and low in U1690 cells, which correlated with the cell surface levels. In spite of the relatively high amount of total DR5, its lower level was detected at the surface of U1285 cells (Figure 1C). While pro-caspase-8 was present in U1285 and U1690 cells, it was not detected in H82, H69, and U1906 cells. All cell lines expressed a relatively high level of pro-caspase-2, most abundantly in U1285 and U1690 cells. No pro-caspase-10 was detected in any of them (Figure 2A). All five cell lines expressed FADD protein (especially U1690). A relatively high level of long and short cFLIP form was apparent in H69, U1690, and U1906 cells. Both cFLIP forms were absent in H82 cells. In U1285 cells, only a low level of cFLIP_L _was detected (Figure 2B). The survivin expression differed significantly among the cell lines, being the highest in H82, U1285, and U1906. A relatively high level of Mcl-1 was found in the SCLC cells studied (except for H69 cells). Bcl-2 protein was detected in 2 out of the 5 cell lines (Figure 2C).

### Doxorubicin and etoposide sensitize SCLC cells expressing caspase-8 protein to TRAIL-induced apoptosis

**Figure 3:**
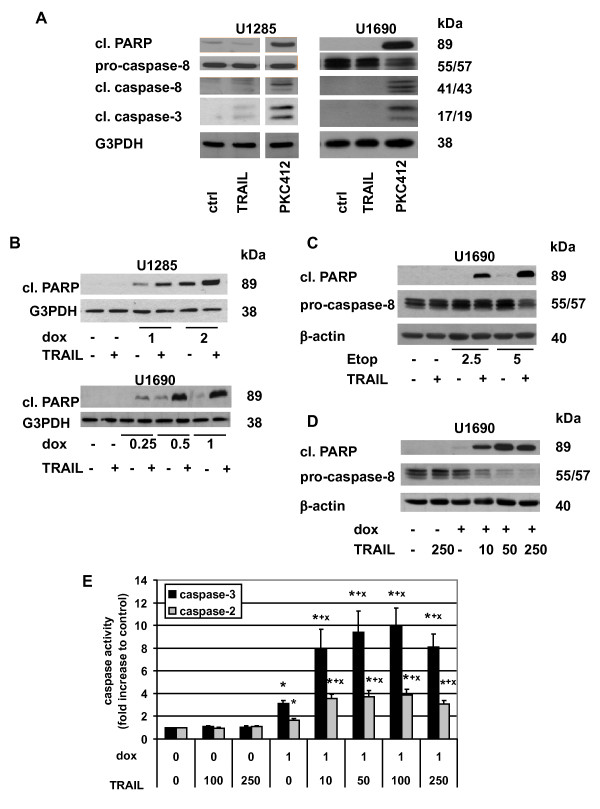
**Characteristic of apoptosis induced by combinatory treatment of doxorubicin/etoposide and TRAIL in U1285 and U1690 SCLC cells**. (A) Cleavage of PARP, caspase-8, and caspase-3 in U1285 and U1690 cells after treatment (24 h) with TRAIL (1000 ng/ml) or PKC412 (1 μM). (B) Cleavage of PARP in U1285 cells treated (24 h) with TRAIL (100 ng/ml), doxorubicin (dox, 1, 2 μM) or their combinations, and in U1690 cells treated (24 h) with TRAIL (100 ng/ml), doxorubicin (0.25, 0.5, 1 μM) or their combinations. (C) Cleavage of PARP and caspase-8 in U1690 cells treated (24 h) with TRAIL (100 ng/ml), etoposide (etop, 2.5 and 5 μM) or their combinations. (D) Cleavage of PARP and caspase-8 in U1690 cells treated (24 h) with TRAIL (10, 50, 250 ng/ml), doxorubicin (1 μM) or their combinations. For (A-D), an equal loading was verified using G3PDH or anti-β-actin antibody. All results are representative of three independent experiments. (E) Caspase-3 and caspase-2 activities (fold increase to control) in U1690 cells treated (24 h) with TRAIL (10, 50, 100, 250 ng/ml), doxorubicin (1 μM) or their combinations (for details see Materials and methods). Results are means ± S.E.M. of three independent experiments. Statistical significance: P < 0.05, (*) versus control, (+) versus TRAIL, (x) versus doxorubicin.

**Figure 4:**
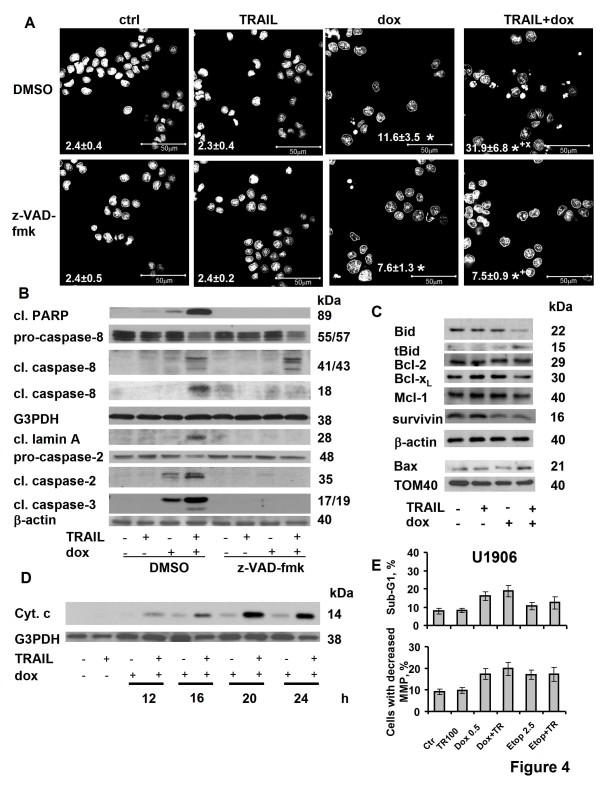
**The role of caspases and mitochondria in apoptosis induced by combination of TRAIL and doxorubicin in U1690 and U1906 SCLC cell lines**. (A) U1690 cells (%) with characteristic apoptotic nuclear morphology (DAPI staining) after pre-treatment (1 h) with z-VAD-fmk (3 μM) or vehicle (DMSO), and subsequent treatment (24 h) with TRAIL (100 ng/ml), doxorubicin (1 μM), or their combination. Results are means ± S.E.M. of three independent experiments. Statistical significance: P < 0.05, (*) versus control, (+) versus TRAIL, (x) versus doxorubicin, (o) versus appropriate sample without z-VAD-fmk. (B) Cleavage of PARP, caspase-8 (12 h) or lamin A, caspase-2, and caspase-3 (24 h) in U1690 cells treated as indicated in (A). An equal loading was verified using G3PDH or anti-β-actin antibody. (C) The cleavage of Bid, level of Bcl-2, Bcl-X_L _or Mcl-1 protein (whole cell lysates), and Bax translocation (mitochondrial fraction) in U1690 cells treated (24 h) with TRAIL (100 ng/ml), doxorubicin (1 μM) or their combination. An equal loading was verified using anti-β-actin (whole cell lysates) or anti-TOM (mitochondrial fraction) antibody. (D) The release of cytochrome *c *into the cytoplasm of U1690 cells treated (12, 16, 20, 24 h) with TRAIL (100 ng/ml), doxorubicin (1 μM) or their combination. An equal loading was verified using anti-G3PDH antibody. Results are representative of three independent experiments. (E) Percentage of U1906 cells with subdiploid DNA content and decreased mitochondrial membrane potential after treatment (24 h) with TRAIL (100 ng/ml), doxorubicin (0.5 μM), etoposide (2.5 μM) or their combination with TRAIL.

Based on the results presented in Figures 1 and 2, two cell lines - U1690 and U1285 - were selected for further investigations. Both expressed pro-caspase-8 protein, an essential molecule for TRAIL apoptotic signaling, and a similar level of cell surface DR5. While we showed a strong induction of apoptosis (caspase-8, -3, and PARP cleavage) in U1690 and U1285 cells treated (24 h) with 1 μM PKC412 (positive control), the cells did not respond even to a relative high dose of TRAIL (1 μg/ml) (Figure 3A). In order to be sensitized to TRAIL-induced apoptosis, cells were co-treated with doxorubicin or etoposide. To determine a suitable concentration of chemotherapeutic drug, the dose response to this agent was investigated using MTS cytotoxicity test (data not shown). Two to three concentrations of the drugs (with low or no cytotoxicity) were then selected to be combined with TRAIL (100 ng/ml). When U1285 cells were simultaneously treated (24 h) with TRAIL and doxorubicin (1 or 2 μM), a dose-dependent potentiation of apoptosis (enhanced PARP and caspase-3 cleavage) was achieved compared to agents used alone (Figure 3B and not shown). Similarly, a strong PARP cleavage was detected upon co-treatment of U1690 cells with TRAIL and doxorubicin (0.5 or 1 μM) as well as TRAIL and etoposide (2.5 or 5 μM) (Figure 3B, C). To investigate the mechanisms involved in sensitization of SCLC cells expressing caspase-8 to apoptosis induced by combined treatment with TRAIL and chemotherapeutic drugs, treatment with doxorubicin was chosen and U1690 cells were subjected to more detailed study. A particular attention was paid to the involvement of caspases, mitochondria, and several important molecules of TRAIL signaling pathway. A substantial cleavage of pro-caspase-8 and PARP was detected in cells co-treated (24 h) with doxorubicin (1 μM) and different doses of TRAIL (10-250 ng/ml) (Figure 3D), together with increase in caspase-2, -3 activity (Figure 3E) and processing (not shown). No changes of caspase-2, -3 activity (compared to control) were detected in cells treated with TRAIL, and a slight enhancement was observed after doxorubicin administration (Figure 3E). Pretreatment with z-VAD-fmk prevented the cooperative apoptotic effect of TRAIL and doxorubicin as demonstrated by inhibition of characteristic apoptotic changes in the nucleus (Figure 4A), and blockage of PARP and lamin A cleavage (Figure 4B). In the presence of z-VAD-fmk, TRAIL- and doxorubicin-induced processing of caspase-2 and -3 was completely blocked. However, the decrease of pro-caspase-8 level, and generation of its 41/43, but not 18 kDa fragments were still detected (Figure 4B).

The combination of TRAIL and doxorubicin induced a significant decrease of full length Bid protein level, accompanied by increased amount of its cleavage product tBid (Figure 4C), a slight decrease of Bcl-X_L_, but no significant changes of Bcl-2 or Mcl-1 protein levels compared to agents used alone (Figure 4C). Significant decrease of the survivin level was also detected in response to doxorubicin alone or its combination with TRAIL (Figure 4C). During 24 h treatment with TRAIL and doxorubicin, an increased translocation of Bax to mitochondria was observed (Figure 4C and not shown), which was associated with an increased percentage of cells with active Bax compared to TRAIL-treated (no Bax activation) or doxorubicin-treated (only low Bax activation) cells (Table 1). The redistribution of cytochrome *c *from mitochondria to cytosol was demonstrated after 12 h (and especially after 16, 20, and 24 h) of incubation with TRAIL and doxorubicin (Figure 4D). Treatment of U1690 cells with TRAIL and doxorubicin also induced significant increase in number of cells with decreased MMP, and with subdiploid DNA content (Table 1). To explore whether combination of doxorubicin or etoposide with TRAIL also exerted a cytotoxic effect in cells lacking caspase-8, the number of U1906 cells with subdiploid amount of DNA and decreased MMP was analyzed following the above mentioned treatments (Figure 4E). As expected, both drugs did not sensitize U1906 cells to TRAIL, demonstrating the requirement of caspase-8 for sensitization of SCLC cells to TRAIL.

**Table 1:** Percentage of U1690 cells with decreased mitochondrial membrane potential, active Bax and subdiploid DNA content.

% of cells	Control	TRAIL	Doxorubicin	TRAIL+Doxorubicin
**Decreased MMP**	2.3 ± 0.6	2.2 ± 0.7	13.4 ± 1.8 *	29.8 ± 6.3 ^*+x^

**Active Bax**	1.5 ± 0.9	2.8 ± 0.4	11.9 ± 3.5 *	44.1 ± 5.3 ^*+x^

**Sub-G1**	4.9 ± 0.6	5.2 ± 0.5	21.9 ± 3.4 ^+^*	40.4 ± 1.7 ^*+x^

Cells were treated (24 h) with TRAIL (100 ng/ml), doxorubicin (1 μM) or their combination. Results are means ± S.E.M. of three independent experiments. Statistical significance: P < 0.05, (*) versus control, (+) versus TRAIL, (x) versus doxorubicin. MMP - mitochondrial membrane potential.

**Figure 5:**
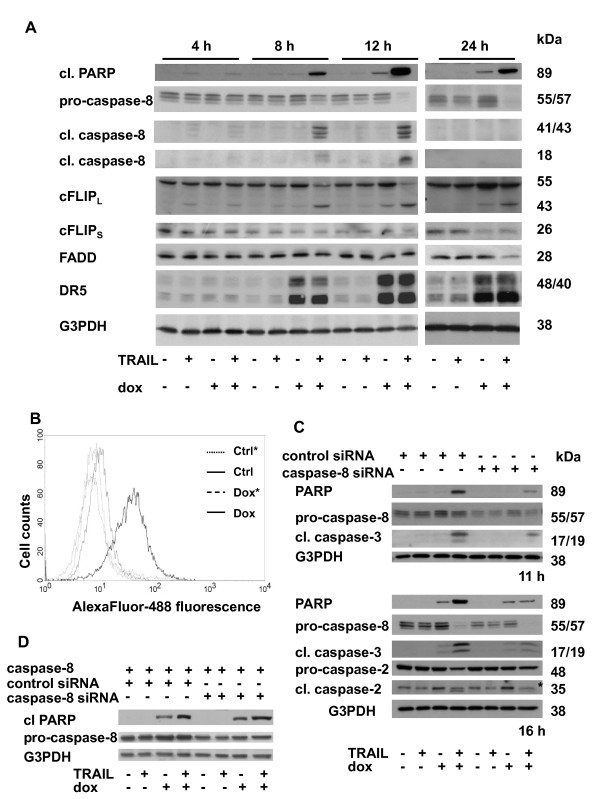
**Time-dependent changes in the expression level of apical proteins involved in TRAIL signaling pathway and an esesential role of caspase-8 in apoptosis induced by combined treatment of doxorubicin and TRAIL in U1690 cells**. (A) Cleavage of PARP, caspase-8, cFLIP_L_, and the level of cFLIP_S_, FADD, and DR5 in U1690 cells treated (4, 8, 12, 24 h) with TRAIL (100 ng/ml), doxorubicin (1 μM) or their combination. An equal loading was controlled using anti-G3PDH antibody. (B) The surface level of DR5 in U1690 cells untreated or treated (24 h) with doxorubicin (1 μM), detected by flow cytometry, using specific primary mouse anti-DR5 antibody, followed by anti-mouse secondary AlexaFluor-488-conjugated antibody. Cells incubated with secondary antibody alone (marked by asterisk) were used to check the background fluorescence. Results are representative of three independent experiments. (C) Cleavage of PARP or caspase-8, -3, -2 in U1690 cells transfected (48 h) with control or caspase-8 siRNA, and then treated (11 or 16 h) with TRAIL (100 ng/ml), doxorubicin (1 μM) or their combination. An equal loading was verified using anti-G3PDH antibody. An asterisk indicated unspecific antibody staining. (D) Cleavage of PARP and level of caspase-8 in U1690 cells transfected (48 h) with control or caspase-8 siRNA, then transfected with pcDNA-MACH alpha 1 (caspase-8) expressing vector (200 ng) and treated (16 h) with TRAIL (100 ng/ml), doxorubicin (1 μM) or their combination. An equal loading was verified using anti-G3PDH antibody.

During the course of 24 h incubation of U1690 cells with TRAIL and doxorubicin, the changes of the expression or cleavage of several important proteins involved in TRAIL signaling were examined. A decrease of pro-caspase-8 level, and accumulation of its 41/43 and 18 kDa fragments were apparent after 8 h, and particularly after 12 h (Figure 5A), accompanied by a significant increase of caspase-8 activity (data not shown) in TRAIL and doxorubicin-treated cells compared to the individual agents. Twenty-four hours of cell incubation with TRAIL and doxorubicin resulted in a complete loss of pro-caspase-8, and disappearance of its specific cleavage fragments due to their rapid degradation. A cleavage of cFLIP_L _to its 43 kDa fragment was detected during 8-24 h of combined treatment, and 12-24 h of doxorubicin administration. A decrease of cFLIP_S _after 12 h, and its almost complete loss after 24 h of incubation with TRAIL and doxorubicin or doxorubicin alone was observed. During the first 12 h, no changes of FADD level were detected after any type of treatment, but 24 h incubation of cells with TRAIL and doxorubicin resulted in significant decrease of FADD. Doxorubicin was responsible for significant up-regulation of the total level of two DR5 protein forms, starting from 8 h in both doxorubicin- or TRAIL and doxorubicin-treated cells (Figure 5A). A significant doxorubicin-induced increase of the surface DR5, but not DR4 was also found using flow cytometry (Figure 5B and not shown). In order to demonstrate a key role of caspase-8, cells were transfected with control or caspase-8 siRNA and then treated (11 or 16 h) with TRAIL and/or doxorubicin. Downregulation of caspase-8 resulted in significant reduction of apoptosis, decreased PARP cleavage and caspase-2, -3 processing in TRAIL and doxorubicin-treated cells (Figure 5C). Markedly, restoration of caspase-8 expression after caspase-8 siRNA-mediated silencing re-sensitized U1690 cells to undergo apoptosis induced by combination of doxorubicin with TRAIL (Figure 5D).

**Figure 6:**
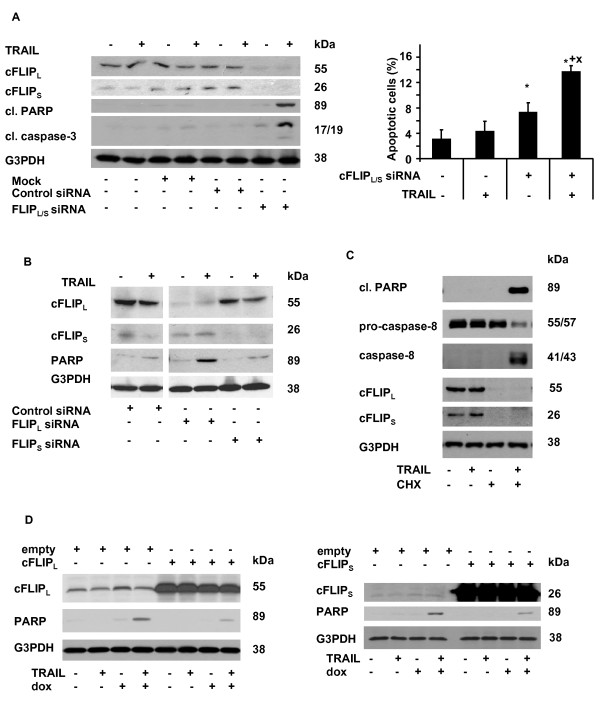
**The role of cFLIP in response of U1690 cells to combined treatment of TRAIL and doxorubicin**. (A) Cleavage of PARP and caspase-3 in U1690 cells non-transfected, mock-transfected (Lipofectamine 2000 only) or transfected (24 h) with FLIP_L/S _or control siRNA, and then treated (24 h) with TRAIL (100 ng/ml). Percentage of cells with apoptotic nuclear morphology after transfection (24 h) with FLIP_L/S _or control siRNAs and subsequent treatment (24 h) with TRAIL (100 ng/ml). Results are means ± S.E.M. of three independent experiments. Statistical significance: P < 0.05, (*) versus control, (+) versus TRAIL, (x) versus doxorubicin. (B) Cleavage of PARP in U1690 cells transfected (24 h) with cFLIP_L_, cFLIP_S _or control siRNA, and then treated (24 h) with TRAIL (100 ng/ml). In (A, B) silencing of cFLIP_L _or cFLIP_S _was verified using anti-cFLIP antibody, and G3PDH was used as a loading control. (C) Cleavage of PARP, caspase-8, and cFLIP_L _or cFLIP_S _level in U1690 cells pre-treated (1 h) with CHX (5 μg/ml), and then treated (12 h) with TRAIL (100 ng/ml). An equal loading was verified using anti-G3PDH antibody. (D) Cleavage of PARP in U1690 cells transfected (24 h) with cFLIP_L_, cFLIP_S _or empty vectors, and then treated (24 or 8 h, respectively) with TRAIL (100 ng/ml). Overexpression of FLIP_L _or cFLIP_S _was controlled using anti-cFLIP antibody, and G3PDH was used as a loading control. Results are representative of three independent experiments.

Several approaches were used to study the role of cFLIP in regulation of TRAIL-induced apoptosis. First, the cells were transfected with siRNA targeting cFLIP_L _and/or cFLIP_S_. Simultaneous elimination of the two cFLIP forms sensitized cells to apoptosis induced by TRAIL (100 ng/ml, 24 h), as demonstrated by enhanced cleavage of PARP, caspase-3, and increase of number of cells with apoptotic nuclear morphology, compared to control siRNA-transfected cells (Figure 6A). To be able to distinguish between the role of cFLIP_L _and cFLIP_S_, another set of experiments was carried out using two siRNAs selectively targetting either cFLIP_L_, or cFLIP_S_. In this case, more intensive TRAIL-induced apoptotic response (increased PARP cleavage) was apparent in cells transfected with cFLIP_L _siRNA (Figure 6B). Second, CHX was used to eliminate cFLIP_L/S _in U1690 cells, which was associated with their sensitization to TRAIL-induced apoptosis shown by enhanced cleavage of PARP and caspase-8 (Figure 6C). Third, the response to TRAIL and doxorubicin was examined following cell transfection with cFLIP_L _or cFLIP_S _vectors. Overexpression of cFLIP_L _or cFLIP_S _rendered cells less sensitive to apoptosis induced by combination of TRAIL and doxorubicin (Figure 6D).

## Discussion

SCLC is a tumour entity where TRAIL monotherapy is not efficient. The loss of some DISC components, associated with inactivation of DR pathway make sensitization of the SCLC cells to TRAIL very difficult [9,10,12,21]. Caspase-8 is frequently silenced in SCLC and other tumours of neuroendocrine origin, usually by aberrant promoter methylation [22,23]. According to our results, 3 out of 5 studied SCLC cell lines were deficient for caspase-8, all lacked caspase-10, and only 1 cell line expressed surface DR4. Previously, restoration of caspase-8 expression by demethylation or gene transfer was shown to sensitize neuroblastoma cells to DR-mediated apoptosis [23]. It has also been suggested that the resistance of SCLC cells lacking caspase-8 to TRAIL might be partially eliminated by combination of demethylation agents and treatment with IFNγ [10]. However additional studies should be performed in order to elucidate how general this phenomenon is.

Notably, we and others found that caspase-8 expressing SCLC cells are also resistant to TRAIL-induced apoptosis. Therefore, we aimed to investigate whether the cytotoxic effects of TRAIL in SCLC could be restored by doxorubicin or etoposide, the conventionally used drugs for treatment of SCLC, and what are the mechanisms responsible for apoptosis resistance of caspase-8 expressing SCLC cells. Our results demonstrated that combination of these drugs with TRAIL might significantly improve efficiency to kill this type of SCLC cells as compared with drugs used alone.

The presence of TRAIL death receptors DR4 and/or DR5 at the cell surface is not always sufficient to trigger apoptosis and that might be due to an existence of the defects of intracellular signaling pathways or high expression of inhibitory proteins. Upregulation of DRs following treatment with chemotherapeutic agents has been demonstrated in some cancer cells, while no effects were apparent in others [24,25]. This upregulation has been shown to be responsible for the synergistic action of TRAIL and chemotherapy in several cell lines [26-28]. In our experiments, untreated U1690 cells expressed a relatively low level of DR5 and no DR4 at the surface. Significant increase of surface DR5, but not DR4 was observed after treatment with doxorubicin, and no significant changes of DcRs level (not shown) as has been demonstrated by others [29]. Our results, therefore, suggest that DR5 plays an exclusive role in mediation of signals triggered by TRAIL in these cells. Furthermore, relocalisation and clustering of DR5 within ceramide-enriched membrane platforms has been reported to affect TRAIL-induced apoptosis in cells treated with doxorubicin [30]. Similar changes at the level of plasma membrane might be also involved in enhancement of TRAIL apoptotic signaling in SCLC cells.

Caspase-8 activation at the DISC proceeds through two subsequent cleavage steps that can be effectively regulated by cFLIP proteins. While cFLIP_L _allows the first cleavage of pro-caspase-8, cFLIP_S _can completely inhibit both of them [31]. cFLIP is known to play an important role in regulation of TRAIL- and chemotherapy-induced apoptosis [20,32,33]. Doxorubicin-induced downregulation of cFLIP_S _contributed to sensitization of prostate cancer cells to apoptotic effects of TRAIL [34,35]. Significant decrease of cFLIP_L/S _level and appearance of specific p43-cFLIP_L _fragment following doxorubicin or TRAIL and doxorubicin treatment indicated their involvement in modulation of apoptotic response of U1690 cells to TRAIL. The role of p43-cFLIP_L _in regulation of caspase-8 activation is still controversial, as both anti- and pro-apoptotic functions of this fragment have been demonstrated [36,37]. CHX-mediated downregulation of cFLIP level previously published also in some other cancer cell types [38,39], resulted in sensitization of U1690 cells to TRAIL-induced apoptosis, showing that resistance of these cells may be at least partially induced by newly synthesized proteins acting upstream of caspase-8. The siRNA-mediated downregulation of cFLIP_L/S _sensitized U1690 cells to TRAIL-induced apoptosis, although to a significantly lesser extent than combination of TRAIL with doxorubicin or etoposide. Moreover, overexpression of cFLIP_L _or cFLIP_S _only partially protected the cells from apoptosis induced by this combinatory treatment. Recently, it has also been shown that silencing of cFLIP induced caspase-8 activation via increased co-localisation of DR5 and ceramide, and significantly enhanced TRAIL-induced apoptosis [40]. Therefore, our results suggest that elimination of cFLIP itself as a single factor may not be sufficient to fully restore the sensitivity to TRAIL-induced apoptosis, and modulation(s) at the level of other molecule(s) such as DR5 need to be involved.

Various chemotherapeutic drugs are known to trigger intrinsic apoptotic pathway [41] and/or increase a susceptibility of mitochondria to apoptotic signals translocated from DRs [25,42,43]. In our experiments we detected an increase of caspase-2 activity that was previously shown as a critical component of DNA damage-induced apoptotic cascade, being activated upstream of mitochondria [44]. Role of caspase-2 as initiator caspase was also observed in apoptosis triggered by other chemotherapeutic drugs [14,45]. In addition to acting as an initiator caspase primarily activated in DR-mediated apoptosis at the DISC level, caspase-8 has also been reported to be activated by other caspases downstream of mitochondria during drug-induced apoptosis [46-48]. This led us to investigate the role of mitochondria and the ordering of caspase activation. Since z-VAD-fmk efficiently blocked TRAIL and doxorubicin-induced cleavage of caspase-2, -3, PARP, lamin A, apoptotic nuclear morphology changes, but not generation of caspase-8 p41/43 kDa fragment, we suggested that the first caspase-8 cleavage step might occur prior to and independently on the activation of other caspases. Moreover, our results demonstrated that siRNA-mediated downregulation of caspase-8 resulted in marked decrease of TRAIL and doxorubicin-induced processing of caspase-2 and -3, and overall apoptosis, while re-expression of caspase-8 fully reverted the apoptotic phenotype. Furthermore, a rapid processing of caspase-8 was a relatively early event in the course of TRAIL and doxorubicin-induced apoptosis compared to attenuated kinetics of mitochondrial events, such as cytochrome *c *release. Therefore, we assume that chemotherapy-mediated modulation of events leading to caspase-8 activation following TRAIL treatment occurs upstream of mitochondria and effector caspases.

As TRAIL by itself did not induce caspase-8 processing, it is likely that the resistance to TRAIL-induced apoptosis is regulated by protein(s) acting upstream of this molecule. Here we demonstrated that doxorubicin was efficient in sensitizing the cells to apoptotic effects of TRAIL that was associated with significant increase of surface and total DR5 level, activation of caspase cascade through processing of caspase-8, specific cleavage of cFLIP_L_, and decrease of cFLIP_S_. Cellular apoptosis was accompanied by cleavage of Bid, Bax activation, decrease of MMP, cytochrome *c *release, decrease of survivin level, and effector caspase activation. Based on the caspase-8 siRNA approach and experiments with z-VAD-fmk we concluded that combination of TRAIL and doxorubicin facilitates caspase-8 processing primarily at the DISC level rather than being secondary result of activation of mitochondrial pathway and/or effector caspases. We suggest the possibility of sensitization of SCLC cells to TRAIL by modulation of the apical events in TRAIL apoptotic signaling at the level of surface DR5 and intracellular inhibitory molecules such as cFLIP.

In summary, although TRAIL monotherapy is completely inefficient in SCLC cells due to defects in initial steps of the DR-mediated pathway, combined treatment with TRAIL and conventional chemotherapeutic drugs, such as doxorubicin and etoposide, might be a promising therapeutic strategy for SCLC expressing caspase-8. Our results showed that doxorubicin and etoposide significantly sensitized these cells to TRAIL-induced apoptosis by modulation of events that facilitate activation of caspase-8. Our study highlights the potential applicability of this combination in chemotherapy of SCLC.

## Abbreviations

BSA: bovine serum albumin; CHX: cycloheximide; DcR: decoy receptor; DISC: death-inducing signaling complex; DR: death receptor; FADD: Fas-associated death domain; FBS: fetal bovine serum; FLIP: FLICE-like inhibitory protein; LC: lung cancer; NSCLC: non-small cell lung carcinoma; PARP: poly(ADP)ribose polymerase; SCLC: small cell lung carcinoma; TNF: tumour necrosis factor; TRAIL: TNF-related apoptosis-inducing ligand.

## Competing interests

The authors declare that they have no competing interests.

## Authors' contributions

AV and VK equally contributed to this work. They were involved in planning and performing experiments, carried out analysis of data and participated in writing of the MS. EJ and OS carried out experiments. BZ participated in the design of the experiments and writing the manuscript. All authors read and approved the final manuscript.
